# Impact of the *c-Myb^E308G^* mutation on mouse myelopoiesis and dendritic cell development

**DOI:** 10.1371/journal.pone.0176345

**Published:** 2017-04-26

**Authors:** Peter Papathanasiou, Sawang Petvises, Ying-Ying Hey, Andrew C. Perkins, Helen C. O’Neill

**Affiliations:** 1 Research School of Biology, Australian National University, Canberra ACT, Australia; 2 Mater Research, Translational Research Institute, University of Queensland, Brisbane QLD, Australia; 3 Clem Jones Research Centre for Regenerative Medicine, Faculty of Health Sciences and Medicine, Bond University, Gold Coast QLD, Australia; Istituto Superiore di Sanità, ITALY

## Abstract

*Booreana* mice carrying the *c-Myb*^*308G*^ point mutation were analyzed to determine changes in early hematopoiesis in the bone marrow and among mature cells in the periphery. This point mutation led to increased numbers of early hematopoietic stem and progenitor cells (HSPCs), with a subsequent reduction in the development of B cells, erythroid cells, and neutrophils, and increased numbers of myeloid cells and granulocytes. Myelopoiesis was further investigated by way of particular subsets affected. A specific question addressed whether *booreana* mice contained increased numbers of dendritic-like cells (L-DC subset) recently identified in the spleen, since L-DCs arise *in vitro* by direct differentiation from HSPCs co-cultured over splenic stroma. The non-lethal *c-Myb* mutation in *booreana* mice was associated with significantly lower representation of splenic CD8^-^ conventional dendritic cells (cDCs), inflammatory monocytes, and neutrophils compared to wild-type mice. This result confirmed the bone marrow origin of progenitors for these subsets since *c-Myb* is essential for their development. Production of L-DCs and resident monocytes was not affected by the *c-Myb*^*E308G*^ mutation. These subsets may derive from different progenitors than those in bone marrow, and are potentially established in the spleen during embryogenesis. An alternative explanation may be needed for why there was no change in CD8^+^ cDCs in *booreana* spleen since these cells are known to derive from common dendritic progenitors in bone marrow.

## Introduction

Hematopoiesis is the generation of fully differentiated blood cells from self-renewing hematopoietic stem cells (HSCs). There are two waves of hematopoiesis in mice: primitive hematopoiesis occurs in the yolk sac from embryonic day 8 (E8) [1], while definitive hematopoiesis is initiated by HSCs residing in the hematogenic endothelium of the aorta-gonado-mesonephros (AGM) region appearing at E10.5 [2–4]. Definitive HSCs migrate to the fetal liver where they expand and differentiate from E12.5 [5]. HSCs then migrate to the bone marrow at E14.5, which becomes the major site for hematopoiesis throughout adult life [5]. HSCs also migrate to the spleen at E14.5, although hematopoiesis in spleen is mostly restricted to the production of erythrocytes [6].

The development of hematopoietic lineages is tightly regulated by transcription factors. Some of these play dual roles in primitive and definitive hematopoiesis, while others are relatively specific to definitive hematopoiesis. For example, *Scl/Tal* and *Gata2* are essential for both primitive and definitive hematopoiesis [7, 8], while *c-Myb* is crucial only for definitive [9]. The *c-Myb* gene encodes a transcription factor that is part of a complex genetic network crucial for maintaining self-renewing hematopoietic stem/progenitor cells (HSPCs) and regulating their differentiation [9]. Most genetic studies of *c-Myb* function have been conducted in mouse models, although most mutations are embryonic lethal [10]. *C-Myb* plays an important role in HSPC self-renewal since conditional knockouts show a loss of stem cells and an accelerated differentiation of hematopoietic progeny [11].

We identified *c-Myb* mutation in a strain called *booreana* (*c-Myb*^*E308G*^: *boo*) by screening for hematopoietic phenotypes in embryos following the mutagenesis of adult sperm with ethylnitrosourea (ENU) [12]. Mice with the *c-Myb*^*E308G*^ mutation were not embryonic lethal, with homozygous *boo/boo* mice surviving to adulthood. An initial analysis of HSPCs in the fetal liver of *boo/boo* compared with wild-type (WT) mice revealed an increase in HSCs with long-term reconstituting capacity (LT-HSCs), multipotential progenitors (MPPs), and common lymphoid progenitors (CLPs) [12]. A more variable effect was seen on common myeloid progenitors (CMPs), with a decrease in granulocyte-macrophage progenitors (GMPs). This was consistent with findings using a c-*Myb*^*M303V*^ mutant strain that showed increased numbers of HSCs, CLPs and CMPs [9]. *Booreana* mice (*c-Myb*^*E308G*^) harbored a single amino acid change in the transactivation domain of *c-Myb* [12]. Like the *M303V* mutation, the *E308G* mutation prevented interaction of the c-MYB protein with its co-activator p300, and led to a complete block in the transactivation capacity of c-MYB and substantial changes in hematopoiesis [9, 13]. An initial study on *booreana* mice showed decreased B lymphopoiesis, increased megakaryopoiesis, and increased numbers of red blood cells, neutrophils and myeloid/dendritic cells (DC) in the blood [12]. Previously, a conditional knockout mouse study indicated a critical role for *c-Myb* in the self-renewal of HSCs and their multi-lineage differentiation [14].

Mice carrying mutations in transcription factor genes have been important in distinguishing lineage relationships between different cell types. Here, we used *booreana* mice to investigate the lineage relationship between dendritic and myeloid subsets. It is generally well established that conventional DCs (cDCs) develop from pre-cDCs [15] that derive from common dendritic progenitors (CDPs) in the bone marrow [16, 17]. Monocytes, on the other hand, develop from CMPs in bone marrow, which then migrate into blood and tissues [18]. However, recent studies identify novel and distinct dendritic and myeloid subsets with unclear lineage origin. For example, monocytes entering tissues were previously thought to differentiate to give tissue macrophages [19]. Recent studies now report their derivation from yolk sac progenitors, which makes them distinct from bone marrow-derived macrophages [20]. These macrophages have been identified in several tissues and include liver Kuppfer cells, epidermal Langerhans cells, and microglia [21–23]. Yolk sac-derived macrophages are F4/80^hi^ and depend on the *PU*.*1* transcription factor for development, while monocytes/macrophages arising from HSPCs in bone marrow are dependent on *c-Myb* [20].

While most mutations in *c-Myb* are embryonic lethal, the single nucleotide mutation *E308G* allows *booreana* mice to survive for several weeks [12]. *Booreana* can therefore be used to measure the impact of *c-Myb* mutation on myelopoiesis in relation to specific cell subsets and help identify their bone marrow origin. We analyzed mutant *c-Myb*^*E308G*^ mice alongside WT mice in terms of numbers of hematopoietic progenitors in bone marrow, and dendritic and myeloid cells in spleen. Since the *in vivo* effects of *c-Myb*^*E308G*^ on hematopoiesis are complex [12], we first checked that the *c-Myb*^*E308G*^ mutational effect was intrinsic only to hematopoietic cells and not somatic cells by comparing the cellular composition in *boo/boo* versus WT chimeras. In addition, *booreana* mice were investigated as a possible source of increased numbers of a rare splenic subset of endogenous dendritic-like cells, namely ‘L-DCs’, that have been previously described by our lab [24, 25]. L-DCs were originally characterized as the progeny of *in vitro* hematopoiesis involving defined subsets of LT-HSCs and MPPs from bone marrow co-cultured above splenic stroma [26–28]. They have since been distinguished as a clear subset within the spleen [24]. *Booreana* mice were therefore investigated as a possible source of higher numbers of the L-DC subset, and perhaps more amenable for study of this rare splenic subset.

## Materials and methods

### Animals

Specific pathogen-free female C57BL/6J (B6: CD45.2) and C57BL/6J.SJL (B6.SJL: CD45.1) mice were bred at the John Curtin School of Medical Research (JCSMR) (Canberra, Australia). *Booreana* (B6: *c-Myb*^*E308G*^) mice were derived in an ENU mutagenesis breeding program involving phenotypic screening of fetal liver cells for early hematopoietic abnormalities [12]. The strain was maintained by heterozygous (*boo/+*) breeding. Progeny were genotyped using PCR to distinguish *c-Myb* and *c-Myb*^*E308G*^ by ear punch DNA in order to identify *wild type* (WT), *boo/+* and *boo/boo* progeny. Animal housing, handling and experimentation procedures were approved by the Animal Experimentation Ethics Committee (Australian National University, Canberra, Australia) under protocol #A2013/11. Animals were sacrificed by cervical dislocation. Pregnant mice for embryo extraction were euthanized using carbon dioxide. Irradiation chimeras were generated by intravenous transfer of 2 x 10^5^ or 1 x 10^6^ fetal liver cells of either *boo/boo* or WT origin (B6; CD45.2) into lethally irradiated (9.5 Gray) B6.SJL (CD45.1) host mice. In addition, mice were given 2 x 10^6^ supporting bone marrow cells of B6.SJL x B6 F_1_ hybrid (CD45.1 x CD45.2) origin to ensure survival.

### Preparation of cells

For analysis of blood composition, whole blood (175 μl) was collected from adult mice into EDTA-treated tubes and run through an ADVIA 120/2120 Hematology System analyser (Siemens AG, Munich, Germany). Cell suspensions of blood, bone marrow and spleen were also prepared as described previously with removal of red blood cells (RBCs) by resuspension in lysis buffer (140mM NH_4_Cl, 17mM Tris base in deionised water). Lineage-negative (Lin^-^) bone marrow was prepared using a lineage depletion cocktail (Miltenyi Biotech, Gladbach, Germany) comprising biotinylated antibodies specific for all hematopoietic lineages (7–4, CD5, CD11b, CD45R, Ly6G/C and Ter119), along with added antibody specific for CD11c to deplete DCs (HL3: Becton Dickinson Pharmingen, San Diego, CA, USA). Column purification of cells using MACS® magnetic bead technology (Miltenyi Biotech) was utilized according to the manufacturer’s protocol. Anti-biotin microbeads in MS or LS columns (Miltenyi Biotech) were used to purify Lin^-^ cells after the column was placed in a magnet (SuperMacs: Miltenyi Biotec) and washing buffer passed through to collect flow-through cells. Splenocytes were also depleted of T and B cells using MACS® magnetic bead technology. Cells were incubated with biotinylated antibodies specific for CD19 (B cells), Thy1.2 (T cells) and Ter119 (RBCs) (eBiosciences, San Diego, CA, USA) prior to separation using anti-biotin microbeads in columns as described above.

### Antibody staining and flow cytometry

Fluorochrome-conjugated antibodies specific for CD11c (N418), CD11b (M1/70), CD115 (AFS98) and streptavidin-APC-Cy7 were obtained from eBiosciences or Bioloegend (San Gabriel, CA, USA). Fluorochrome-conjugated antibodies specific for CD8α (53–6.7), B220 (RA3-6B2), MHC-II (AF6-120.1), CD3ε (145-2C11), c-kit (2B8), CD16/32 (Clone 93), Gr-1 (RB6-8C5), Sca1 (E13-161.7), CD34 (RAM34), IL-7Rγ (A7R34), Flt3 (A2F10), CD150 (TC15-12F12.2), Ly6C (HK1.4), Ly6G (1A8), streptavidin-PE-Cy7, streptavidin-PE and streptavidin-FITC were obtained from Biolegend. Goat-anti rat-PE-Texas Red was obtained from Invitrogen (Eugene, OR, USA). Isotype control antibodies including Rat IgG_2a_-FITC (R35-95), Rat IgG_2b_-PE (RTK4530), Rat IgG_2b_-PE-Cy7 (eB149/10H5), Mouse IgG_2a_-biotin (eBM2a) and Hamster IgG-APC (eBio299Arm) were obtained from eBiosciences.

Cells were stained as previously described [24, 28]. Briefly, 1 x 105–1 x 10^6^ cells were incubated with purified CD16/32 antibody (Clone 93; eBiosciences) for 15 min to block surface Fc receptors. Cells were then washed with DMEM/1%FCS/0.1%NaN_3_ and stained with primary antibodies for 20 min on ice. Any secondary reagents were added to the stained cells after a washing step, and further incubated for 20 min on ice. Cells were washed twice and resuspended in DMEM/1%FCS/0.1%NaN_3_ for flow cytometric analysis using a LSRII flow cytometer (Becton Dickinson). Cells were stained with PI (1μg/ml) for live cell discrimination. Gates were set to delineate cell subsets using isotype control antibodies and ‘fluorescence minus one’ controls. Cell subset analysis was performed using BD FACSDiva Software (Becton Dickinson) and FlowJo Software (Tristar; Phoenix, Arizona, USA).

### Analysis of hematopoietic progenitors

Lin^-^ bone marrow was prepared as described above and then stained with antibodies for delineation of progenitors. The lineage depletion cocktail of antibodies (Miltenyi) supplemented with antibody to CD11c, was used to identify Lin^-^ cells. Staining for Sca-1 and c-kit was used to delineate the Lin^-^Sca-1^+^ckit^+^ (LSK) subset [29]. LT-HSCs were then identified as the CD150^+^Flt3^-^ subset of LSK cells [30, 31]; MPPs as CD150^-^Flt3^+^ LSK cells [32]; macrophage dendritic cell progenitors (MDPs) as Lin^-^Sca-1^-^c-kit^hi^Flt3^+^CD115^+^ cells [33], and CDPs as Lin^-^Sca-1^-^c-kit^lo^Flt3^+^CD115^+^ cells [16, 17]. Cells were analysed by flow cytometry, isotype control antibodies used to set gates and propidium iodide (PI: 1 μg/mL) staining of cells was used for dead cell discrimination.

### Establishment of co-cultures over splenic stroma

Cells were cultured as described previously [28] in Dulbecco’s Modified Eagles Medium (DMEM) supplemented with 4 g/L D-glucose, 6 mg/L folic acid, 36 mg/L L-asparagine, 116 mg/L L-arginine, to which was added 10% fetal calf serum, 10mM HEPES, 2 mM L-glutamine, 100 U/L penicillin, 100 μg/L streptomycin, and 5 x 10^−5^ M 2-mercaptoethanol. The splenic stromal cell line 5G3 was passaged every 4 days by scraping and transferring non-adherent cells to a new flask [34]. Cells were maintained in 5% CO_2_ in 95% humidity at 37°C.

For establishment of co-cultures, Lin^-^ bone marrow (10^4−5^ cells/ml) was overlaid on to near-confluent 5G3 stroma in replicate 25 cm^2^ flasks (5 ml cultures). This procedure has been described in detail previously [27, 28, 34, 35]. Medium change was performed every 3–4 days by replacement of 2.5 ml medium. Non-adherent cells were collected at days 14, 21, and 28 for analysis of dendritic and myeloid cells produced using antibody specific for CD11c, CD11b, CD8α, B220 and MHC-II and flow cytometry.

### Statistical analysis

Data were analyzed using the two-tailed Student’s *t*-test with significance determined at p ≤ 0.05. For experiments involving only 3 or 4 replicates, significance was determined using the Wilcoxon Rank Sum Text (p ≤ 0.05).

## Results

### Mutational effect of *c-Myb*^*E308G*^ is intrinsic to hematopoietic cells

Although *booreana* mice show multiple changes amongst hematopoietic cells, the mutation is not lethal, with no loss of essential hematopoietic subsets [12]. In order to determine whether the mutant phenotype was intrinsic to hematopoietic cells as opposed to an indirect or somatic effect, fetal liver cells of *boo/boo* and WT mice were compared for their capacity to reconstitute the hematopoietic compartment of chimeras. Lethally irradiated B6.SJL (CD45.1) mice were reconstituted with *boo/boo* or WT fetal liver of B6 (CD45.2) origin, along with supporting bone marrow cells from B6 x B6.SJL (CD45.2 x CD45.1) mice (S1 Fig). Two doses of test cells were transplanted and reconstitution monitored by flow cytometric analysis of antibody-stained peripheral blood leukocytes at 4-week intervals. The staining and gating procedure used to analyze leukocyte subsets is shown in S2 Fig. The ADVIA machine was also used to monitor cell composition of blood at 16 weeks.

**Fig 1:**
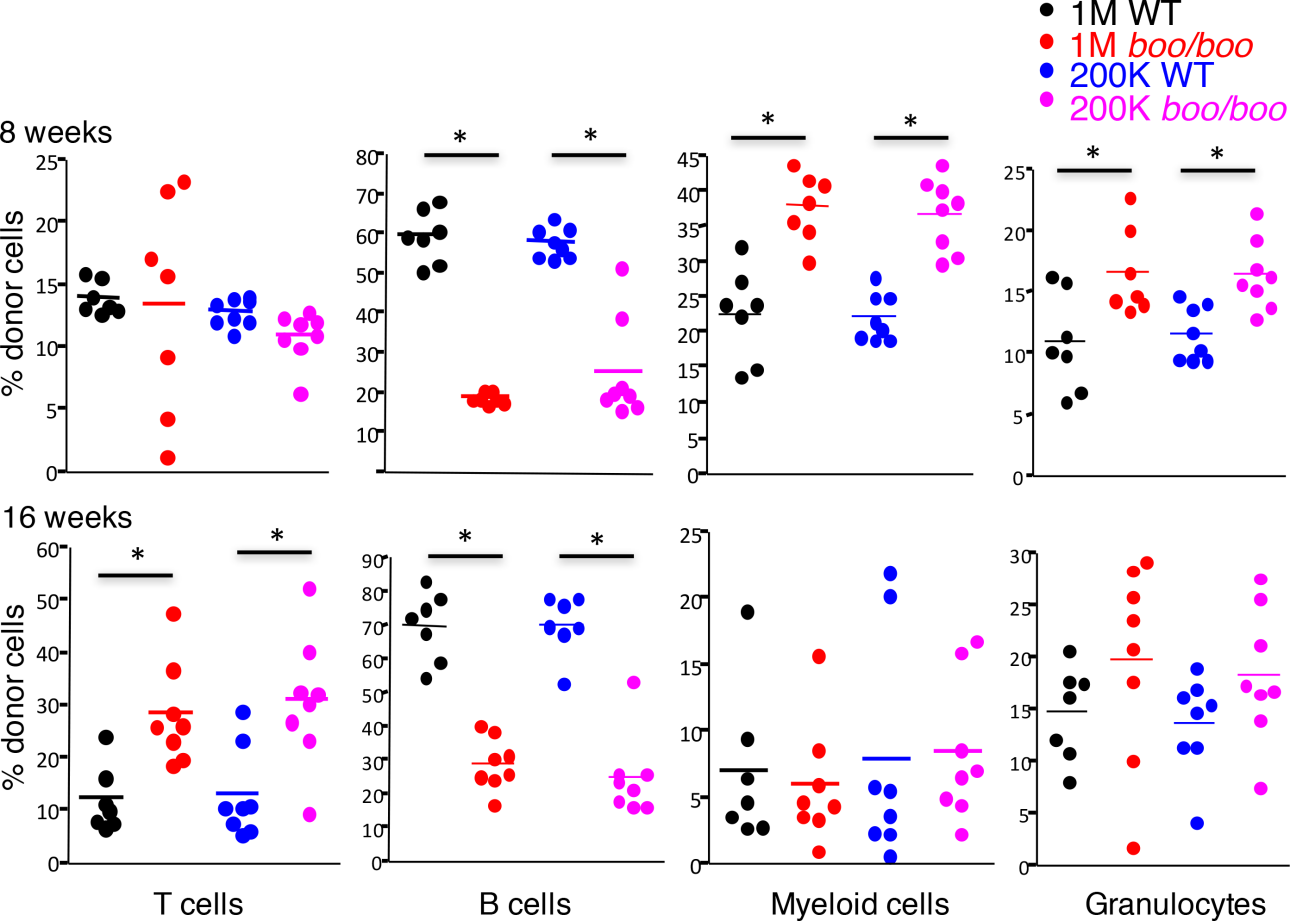
*In vivo* reconstitution potential of *booreana* fetal liver HSCs. Chimeras were generated to investigate the *in vivo* reconstitution ability of *booreana* (*boo*) versus WT fetal liver. Analyses involved a quantitative and kinetic study of the lineage potential of 1 x 10^6^ (1M) or 2 x10^5^ (200K) fetal liver cells from *boo/boo* and WT mice of CD45.2 origin transplanted into lethally irradiated CD45.1 mice. Donor cell reconstitution was assessed via flow cytometric analysis of subsets in peripheral blood of chimeras at 8 and 16 weeks post reconstitution. The proportional representation of T cells (CD3^+^), B cells (B220^+^), myeloid cells (CD11b^+^) and granulocytes (Gr1^+^) was measured within the donor-derived (CD45.2) leukocyte compartment of peripheral blood. Individual data points are shown, with mean value indicated by a horizontal bar. All comparisons between equivalent *boo/boo* and wild type chimeras were significantly different (p ≤ 0.05) except for T cells at 8 weeks, and macrophages and granulocytes at 16 weeks. More complete data at 4, 8, 12 and 16 weeks are summarised in S3 Fig. Significantly different data sets are shown by *.

A relative increase in myeloid cells and granulocytes compared with lymphoid cells was characteristic of *booreana* mice. This was evident in the peripheral blood of *boo/boo* chimeras compared with WT chimeras at 4, 8 and 12 weeks, although this increase was lost by 16 weeks (Fig 1, S3 Fig). The early increase in myeloid cell numbers was balanced by a compensatory reduction in B cell numbers but not T cell numbers, which reflected multiple changes in cell production in the bone marrow (Fig 1, S3 Fig). After 16 weeks, *boo/boo* chimeras showed increased numbers of T cells in peripheral blood, reduced numbers of B cells, and equivalent myeloid cell numbers compared to WT chimeras. The increase in CD11b^+^ myeloid cells out to 12 weeks could reflect an increase in both precursors and mature cells of the dendritic and macrophage/monocyte lineages emanating from higher numbers of bone marrow progenitors. A higher proportion of Gr1^+^ cells out to 12 weeks could reflect increased numbers of neutrophils and other granulocytes entering the peripheral blood from bone marrow. These results indicated an important stimulatory role for *c-Myb*^*E308G*^ in myelopoiesis, with compensatory changes in T and B cell numbers. Since significant changes were identified in the mature hematopoietic cell populations of *boo/boo* chimeras compared to WT chimeras, it was clear that the *c-Myb*^*E308G*^ mutant phenotype was intrinsic to the hematopoietic compartment and was not the result of an indirect change related to the microenvironment in which HSCs develop. The mutation was not lethal and did not result in the complete loss of one or more hematopoietic subsets.

**Fig 2:**
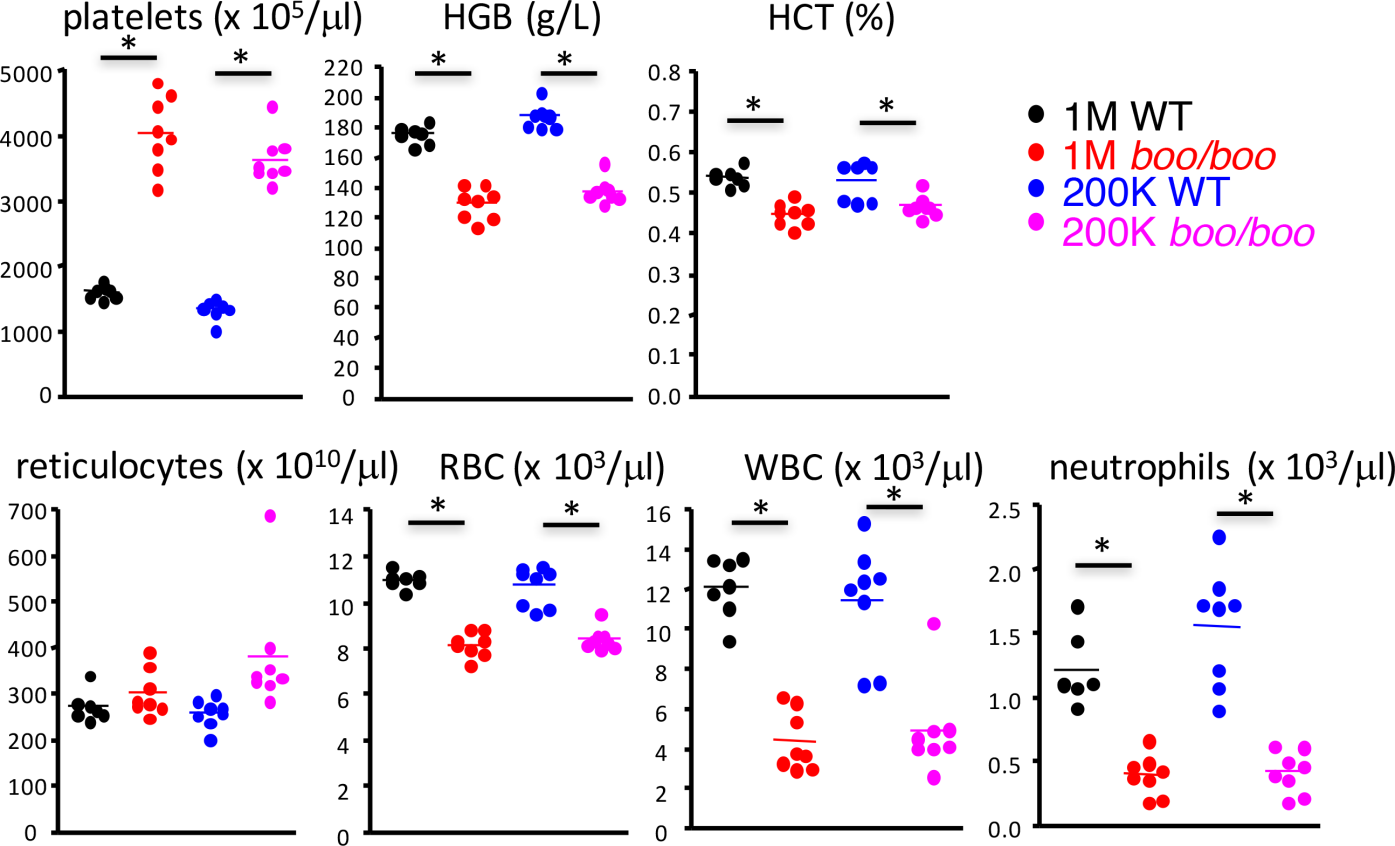
Peripheral blood analysis of chimeras reconstituted with *booreana* fetal liver. Chimeras were prepared as described in Fig 1. At 16-weeks post-transplant, blood composition was analyzed. Individual data points are shown for *boo/boo* and WT chimeras, with the mean value indicated by a horizontal bar. All comparisons between equivalent *boo/boo* and wild type chimeras were significantly different (p ≤ 0.05), except for reticulocyte counts in chimeras reconstituted with 1 x 10^6^ cells. HGB = hemoglobin, HCT = hematocrit, RBC = red blood cells, WBC = white blood cells. Significantly different data sets are shown by *.

Quantitation of cells in the blood of 16 week chimeras revealed that *boo/boo* chimeras had significantly fewer white blood cells and neutrophils than WT chimeras (Fig 2). The average number of these cells was reduced by ~3-fold due to the *c-Myb*^*E308G*^ mutation. A 2-fold increase in platelet numbers in *boo/boo* chimeras over WT chimeras was evident, along with a reduced production of mature red blood cells, but not reticulocytes. Consistent with this observation was a reduction in hematocrit and hemoglobin levels (Fig 2). Blood analysis was consistent with a reduction in both erythyropoiesis and granulopoiesis in *boo/boo* chimeras after 16 weeks.

### *Booreana* bone marrow contains more hematopoietic progenitors

**Fig 3:**
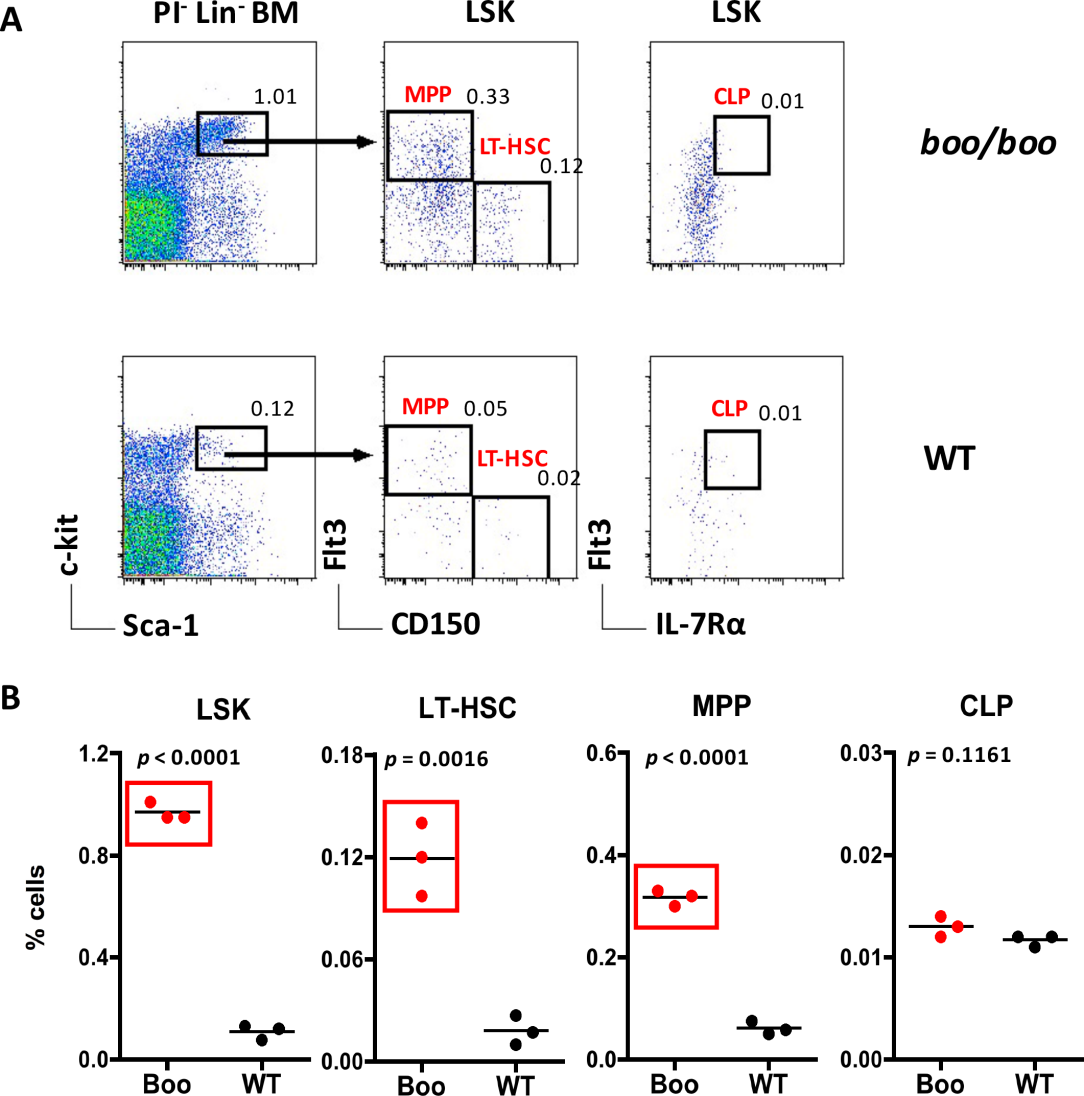
Analysis of bone marrow progenitors in *booreana* (*boo/boo*) and WT mice. Bone marrow from adult mice was prepared and stained with antibodies to distinguish hematopoietic progenitors flow cytometrically. A lineage cocktail of antibodies was used to gate Lin^-^ subsets, and Sca-1 and c-kit staining used to identify the Lin^-^c-kit^+^Sca-1^+^ (LSK) subset. Staining for Flt3 and CD150 was used to distinguish LT-HSCs and MPPs, and staining for IL-7R and Flt3 was used to distinguish CLPs. Propidium iodide (PI; 1 μg/ml) staining was used to gate live cells as PI^-^. Gates were set on bivariate plots using isotype control antibodies, and numbers in gates reflect % positive cells amongst Lin^-^PI^-^ bone marrow cells. **(A)** Staining profiles for representative individual mice are shown. **(B)** Percent cells amongst viable Lin^-^ bone marrow is shown for 3 individual mice, with mean shown by a bar and statistically significant results (p ≤ 0.05) boxed in red.

Since the *c-Myb*^*E308G*^ mutation effects myelopoiesis, we compared the bone marrow of adult *booreana* mice with WT mice for known HSPC subsets. Bone marrow cells were isolated, stained with antibody cocktails and viable (PI^-^) cells analysed flow cytometrically to delineate subsets. Gates were used to detect Lin^-^ cells, and then the subset of Lin^-^Sca1^+^ckit^+^ (LSK) enriched for HSCs was identified. Further analysis employing three more markers–CD150, Flt3, IL-7Rα –facilitated the gating of LT-HSCs, MPPs and CLPs (Fig 3A). Results shown in Fig 3B reveal that bone marrow has a statistically significant increase in the number of LT-HSCs and MPPs in *boo/boo* mice over WT mice, consistent with previous findings for fetal liver [12]. Representation of CLPs in bone marrow was similar for *boo/boo* and WT mice, a result which differed from previous fetal liver findings [12]. A 12-fold increase in the percentage of LT-HSCs in bone marrow is reflective of the *booreana* phenotype and consistent with *c-Myb*^*E308G*^ impacting HSC self-renewal and proliferation.

**Fig 4:**
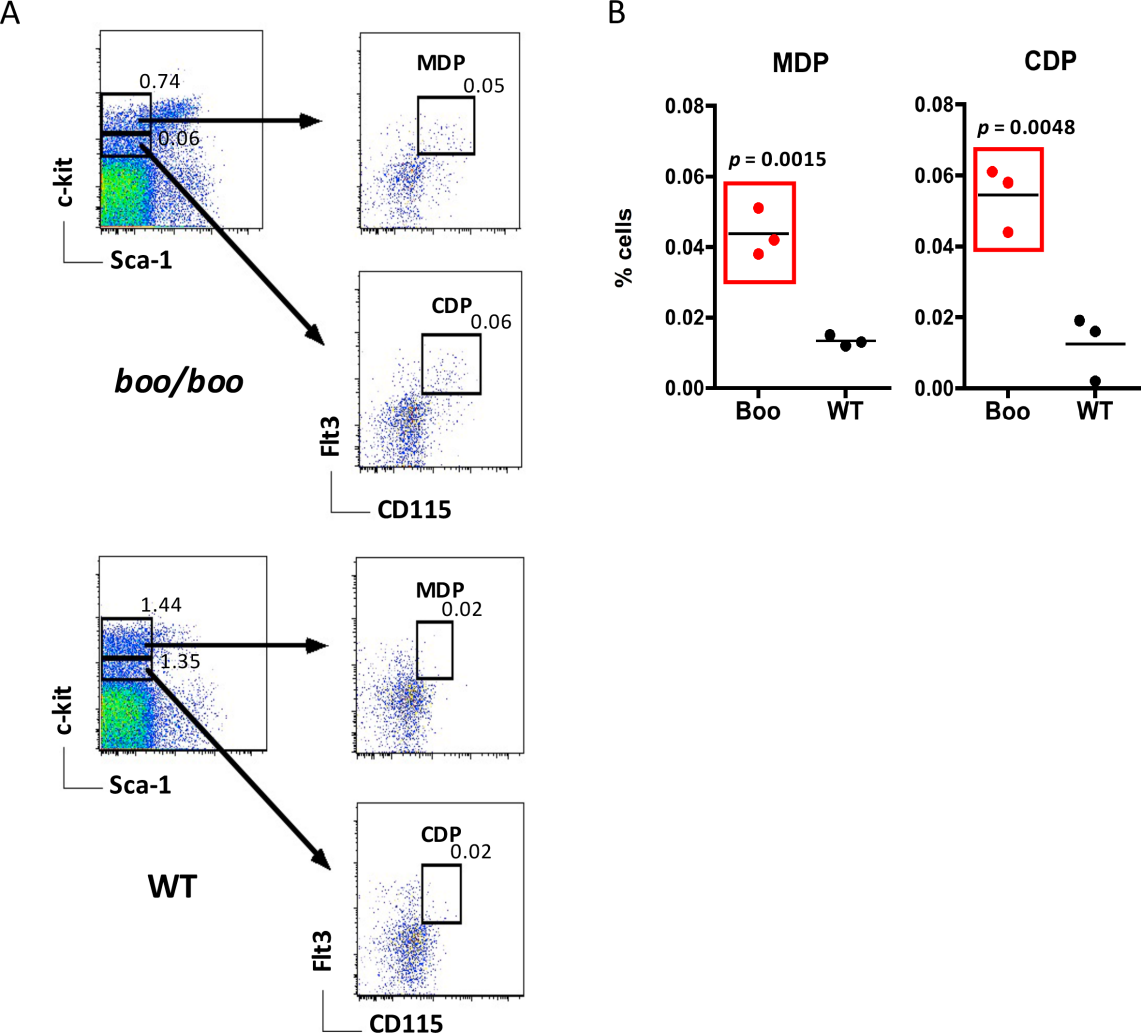
*Booreana* (*boo/boo*) mice have increased numbers of dendritic cell progenitors in bone marrow. Cells from *boo/boo* and WT mice were prepared and stained with antibodies to distinguish macrophage dendritic cell progenitors (MDP) and common dendritic cell progenitors (CDP) flow cytometrically. A lineage cocktail of antibodies was used to gate the Lin^-^ cells and so exclude mature cells. Sca-1 and c-kit staining was used to identify the Lin^-^c-kit^hi^Sca-1^-^ and Lin^-^c-kit^lo^Sca-1^-^ subsets, and staining for Flt3 and CD115 used to distinguish MDP and CDP amongst these subsets. Propidium iodide (PI; 1 μg/ml) staining was used to gate live cells (PI^-^). Gates were set on bivariate plots using isotype control antibodies, and numbers in gates reflect % positive cells amongst Lin^-^PI^-^ bone marrow cells. **(A)** Profiles for representative individual mice are shown. **(B)** Percent cells amongst viable Lin^-^ bone marrow is shown for 3 individual mice, with mean value shown as a bar, and statistically significant results (p ≤ 0.05) boxed in red.

In terms of dendritic and myeloid cell progenitors, the more recently defined CDP and MDP subsets in bone marrow were investigated since these were found to be responsible for repopulation of dendritic and myeloid subsets in spleen [11, 36]. Lin^-^ bone marrow cells were first gated, and the Sca1^-^ subset divided on the basis of c-kit expression. The c-kit^hi^ subset was further divided by expression of Flt3 and CD115 to delineate the described MDP population [33], while the c-kit^lo^ subset of Flt3^+^CD115^+^ cells was gated to represent CDPs [11] (Fig 4A). Both *boo/boo* and WT bone marrow contained subsets of MDPs and CDPs, although *boo/boo* mice showed a significantly higher proportion of both progenitor subsets (Fig 4B), which was in line with higher numbers of the earlier HSC and MPP subsets (Fig 3B). This increase suggested higher potential for myelopoiesis and DC development in the bone marrow of *boo/boo* mice, and was consistent with increased myelopoiesis in the peripheral blood of chimeras out to 12 weeks (Fig 1).

### Reduced numbers of late myeloid progenitors in *booreana* bone marrow

**Fig 5:**
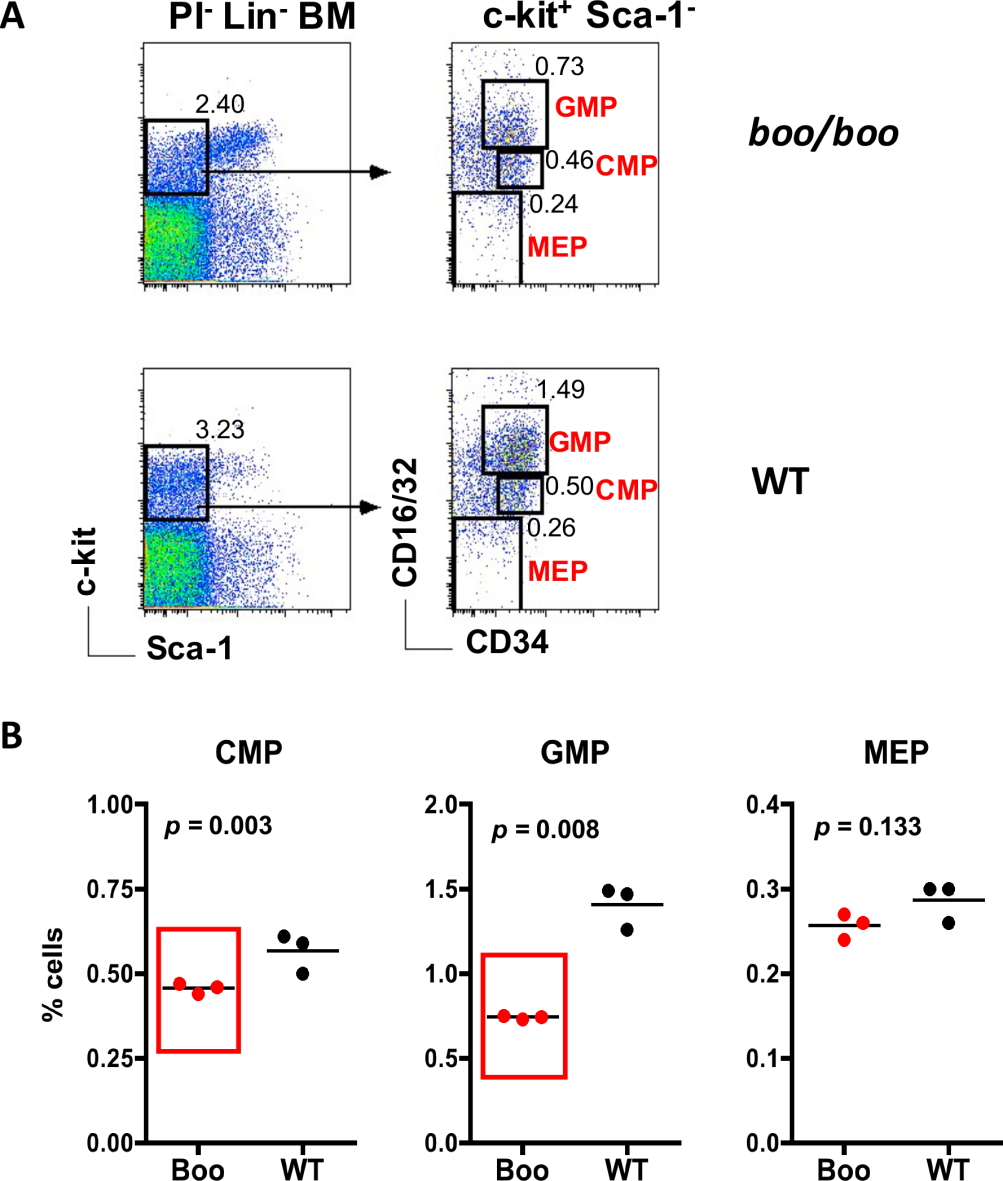
*Booreana* (*boo/boo*) mice show a reduction in myeloid progenitors in bone marrow. Cells from *boo/boo* and WT mice were prepared and stained with antibodies to distinguish hematopoietic progenitors flow cytometrically. A lineage cocktail of antibodies was used to gate the Lin^-^ subsets, so excluding mature cells. Sca-1 and c-kit staining was used to identify the Lin^-^c-kit^+^Sca-1^+^ subset. Staining for CD34 and CD16/32 was used to distinguish CMPs, GMPs, and MEPs. Propidium iodide (PI; 1 μg/ml) staining was used to gate live cells (PI^-^). Gates were set on bivariate plots using isotype control antibodies, and numbers in gates reflect % positive cells amongst Lin^-^PI^-^ bone marrow cells. **(A)** Profiles for representative individual mice are shown. **(B)** Percent cells amongst viable Lin^-^ bone marrow is shown for 3 individual mice, with mean value shown as a bar, and statistically significant results (p ≤ 0.05) boxed in red.

Oligopotent late myeloid progenitors have been described as able to differentiate to give several myeloid lineages [37]. The frequency of the described CMPs, GMPs, and myelo-erythroid progenitors (MEP) was therefore compared in *boo/boo* and WT bone marrow. Lin^-^ bone marrow was gated, followed by delineation of Sca-1^-^ cells with high c-kit expression. This subset was then divided on the basis of CD34 and CD16/32 expression to give CMPs, GMPs, and MEPs (Fig 5A). *Boo/boo* mice showed a slight but significant reduction in CMPs, and a significant 2-fold reduction in GMPs in relation to WT mice (Fig 5B). These results contrasted sharply with the increased representation of earlier progenitors in *boo/boo* mice (Fig 3). The proportion of MEPs did not differ significantly between *boo/boo* and WT mice, a result consistent with only minor changes in RBCs, reticulocyte and hemacrit counts in *boo/boo* compared with WT chimeras (Fig 2). However, previous studies on fetal liver showed lower numbers of GMPs and MEPs in *boo/boo* mice compared with WT mice [12].

### Effect of *c-Myb*^*E308G*^ on myelopoiesis in spleen

**Fig 6:**
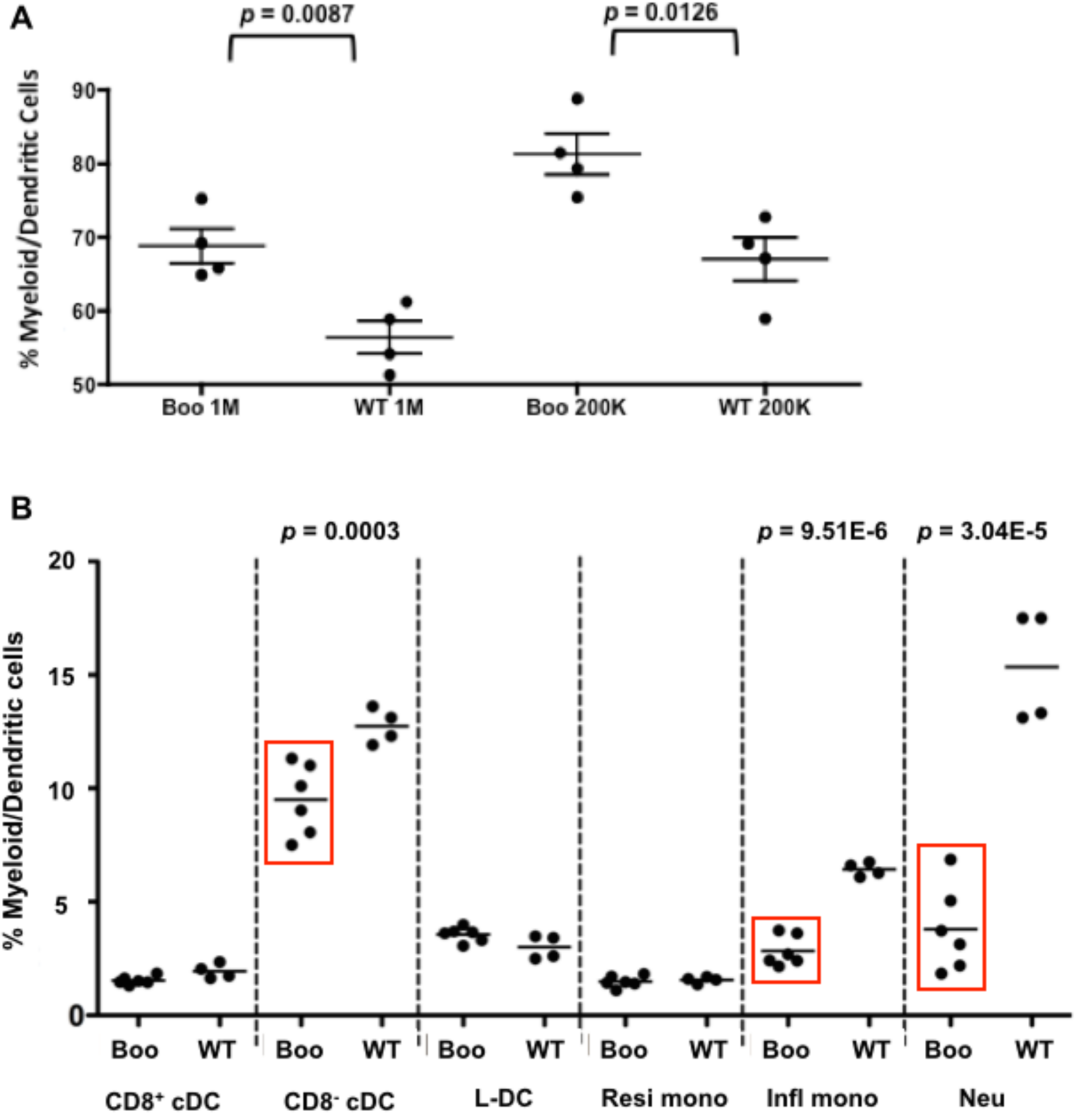
Analysis of splenic myeloid and DC subsets in *booreana* versus WT mice. **(A)** Chimeras were prepared as described in Fig 1 and sacrificed at 52 weeks. The total splenic dendritic and myeloid subset was purified via red blood cell lysis and T/B cell depletion. Cells were stained with antibody to detect CD45.2^+^ cells, and the population gated as CD11b^+^ and/or CD11c^+^ to estimate % dendritic/myeloid cells, respectively. Data represent mean ± SE (*n* = 4), and *p* values for statistically significant pairs of data shown. **(B)** Spleens of adult *booreana* and WT mice were enriched for DC and myeloid cells via red blood cell lysis and T/B cell depletion. Cells were then stained with antibodies to delineate subsets of CD8^+^ cDCs (CD11c^hi^CD11b^-^MHCII^+^CD8^+^), CD8^-^ cDCs (CD11c^hi^CD11b^lo^MHCII^+^CD8^-^), L-DCs (CD11c^lo^CD11b^hi^Ly6C^-^Ly6G^-^), resident monocytes (resi mono: CD11b^hi^CD11c^lo/-^Ly6C^+^Ly6G^-^), inflammatory monocytes (infl mono: CD11b^hi^CD11c^-^Ly6C^hi^Ly6G^-^), and neutrophils (neu: CD11b^hi^CD11c^-^Ly6C^+^Ly6G^+^). The proportional representation of each subset was calculated relative to the total dendritic and myeloid population in spleen (i.e. CD11b^+^ and/or CD11c^+^ cells, respectively). Data (mean) are shown for *boo/boo* (*n* = 3) and WT (*n* = 4) mice. Statistically significant findings (*p* ≤ 0.01) are boxed and *p* values are shown.

We next investigated the impact of the *c-Myb*^*E308G*^ mutation on the development of individual dendritic and myeloid subsets in spleen. In particular, the impact of *Myb*^*E308G*^ on L-DC production was of interest given the discovery that this novel subset develops from hematopoietic progenitors endogenous to spleen [38]. Initially, the total population of myeloid and dendritic cells (CD11c^+^ and/or CD11b^+^) was quantitated in spleens of *boo/boo* and WT chimeras at 52 weeks post reconstitution. The production of these chimeras was described in S1 Fig and S2 Fig. Overall, *boo/boo* chimeras showed a significant 2-fold greater population of dendritic/myeloid cells in spleen than did WT chimeras (Fig 6A). While *boo/boo* and WT chimeras showed equivalent representation of myeloid cells and granulocytes in blood at 16 weeks (Fig 1), *boo/boo* chimeras showed higher numbers than WT chimeras after 52 weeks. Over the long-term, *c-Myb*^*E308G*^ had the effect of increasing myelopoiesis.

An investigation of spleen in adult mutant mice is possible since the *c-Myb*^*E308G*^ mutation is not embryonically lethal and *boo/boo* mice live to adulthood. Further studies therefore investigated the effect of *c-Myb*^*E308G*^ on the representation of individual dendritic and myeloid subsets in spleen. Individual mice were analyzed, and cells stained to detect mature subsets of CD8^+^ cDCs (CD11c^hi^CD11b^-^MHCII^+^CD8^+^) and CD8^-^ cDCs (CD11c^hi^CD11b^lo^MHCII^+^CD8^-^). In a separate staining, subsets of L-DCs (CD11c^lo^CD11b^hi^Ly6C^-^Ly6G^-^), resident monocytes (CD11b^hi^CD11c^lo/-^Ly6C^+^Ly6G^-^), inflammatory monocytes (CD11b^hi^CD11c^-^Ly6C^hi^Ly6G^-^) and neutrophils (CD11b^hi^CD11c^-^Ly6C^+^Ly6G^+^) were also quantitated within the total spleen population of dendritic and myeloid cells. Of the DC subsets, only CD8^-^ cDCs were found to be significantly reduced in number in *boo/boo* compared with WT mice. Among the myeloid subsets, both inflammatory monocytes and neutrophils were significantly reduced, but not resident monocytes. The representation of CD8^+^ cDCs and L-DCs did not differ between *boo/boo* and WT mice. The mutational effect of *c-Myb*^*E308G*^ on subset size served to distinguish L-DC from CD8^-^ cDCs, and inflammatory monocytes from resident monocytes, consistent with a distinct lineage origin for these subsets.

The increased representation of CD11c^+^ and/or CD11b^+^ cells in peripheral blood (Fig 1) and spleen (Fig 6A) of *boo/boo* chimeras was not supported by an increase in the representation of carefully defined mature dendritic and myeloid cells in *boo/boo* adult spleen (Fig 6B). Measurement of CD11b^+^ cells in blood (Fig 1), and of CD11c^+^/CD11b^+^ cells in spleens of *boo/boo* chimeras (Fig 6A) includes precursors pouring out of bone marrow into blood and spleen, and will not accurately reflect numbers of mature cells in these organs. Increased myelopoiesis in *boo/boo* mice would therefore appear to reflect the production of more immature myeloid cells. Production of mature cells in spleen would be limited by available microenvironments and competition for empty niche spaces. The production of CD8^+^ cDCs, L-DCs, and resident monocytes was unaffected by *c-Myb*^*E308G*^, while the production of inflammatory monocytes, neutrophils, and CD8^-^ cDCs was reduced.

### Assessment of L-DC progenitors in bone marrow

Previous studies have shown that the spleen contains progenitors of L-DCs that seed spleen stromal co-cultures to produce this novel dendritic-like subset [39–41]. A number of studies now equate L-DC progenitors with LT-HSC and MPP subsets in bone marrow, fetal liver, and spleen [26, 28, 38]. In order to compare the prevalence of L-DC progenitors in *boo/boo* versus WT mice, Lin^-^ bone marrow was prepared and seeded into splenic stromal co-cultures as previously described [27, 34, 35]. To avoid the influence of environment on development of L-DC progenitors, bone marrow was isolated from chimeras described in Fig 1 and S1 Fig. Cells were sorted on the basis of lineage and CD45.2 expression to yield Lin^-^ bone marrow of *boo/boo* and WT origin from individual chimeras, each having a CD45.1 WT background (S1 Fig). Equal numbers of cells were then co-cultured above 5G3 stroma, and the relative capacity of *boo/boo* and WT bone marrow progenitors was compared for myelopoiesis and L-DC production.

**Fig 7:**
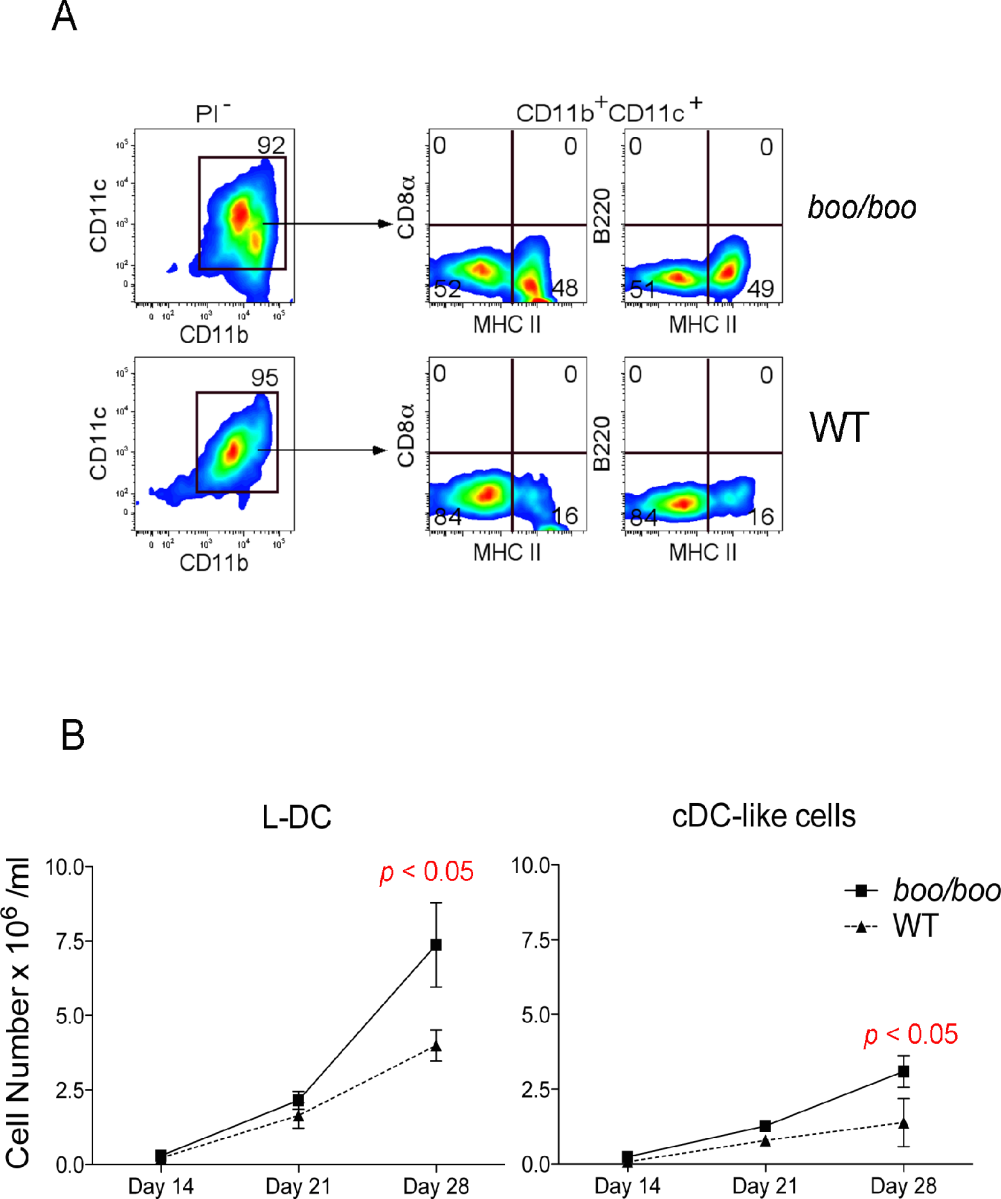
Hematopoiesis due to *booreana* (*boo/boo*) and WT bone marrow in co-cultures. Bone marrow was prepared from one-year old chimeras described in Fig 1. Donor-derived CD45.2^+^ bone marrow cells were sorted as a Lin^-^ population of *boo/boo* or WT origin, and equal numbers co-cultured over 5G3 splenic stroma. Individual co-cultures were established from 4 *boo/boo* chimeras and 2 WT chimeras. Cell production was monitored over time by staining non-adherent cells using antibodies to CD11c, CD11b, MHC-II, and CD8α, or with isotype controls. Propidium iodide (PI; 1 μg/ml) staining was used to gate live cells (PI^-^). Gates were set on bivariate plots using isotype control antibodies, and numbers shown in gates reflect % positive cells. **(A)** Data shows staining of 21 day co-cultures established from one of each chimera type. L-DCs were gated as CD11c^lo^CD11b^hi^MHC II^-^CD8α^-^B220^-^ cells, and cDC-like cells as CD11c^hi^CD11b^lo^MHC II^+^CD8α^-^B220^-^. **(B)** Cell production was calculated and shown as mean ± SE for co-cultures established from *boo/boo* (*n* = 4) and wild-type (WT) (*n* = 2) chimeras. Significantly different data pairs are indicated.

Non-adherent cells were collected over time and stained with specific antibodies for the identification and quantitation of cell subsets using flow cytometry as previously described [27, 28]. Co-cultures established with both *boo/boo* and WT Lin^-^ bone marrow were each produced greater numbers of L-DC (CD11b^+^CD11c^+^MHC-II^-^CD8^-^B220^-^) than cDC-like cells (CD11b^+^CD11c^+^MHC-II^+^CD8^-^B220^-^) (Fig 7A); these are the two subsets known to be produced in Lin^-^ bone marrow co-cultures over 5G3 [27]. After 28 days, co-cultures established with *boo/boo* Lin^-^ bone marrow yielded significantly higher numbers of both L-DC and cDC-like progeny than WT (Fig 7B). It was previously found that the L-DC progenitor was contained within subsets of LT-HSCs and MPPs [28], while the progenitor of cDC-like cells was contained within CDP and MDP subsets [28]. These results are consistent with evidence for higher numbers of these progenitors in *boo/boo* over WT bone marrow (Figs 3B and 4B).

As with all previous 5G3 co-culture studies involving overlaid hematopoietic progenitors, only myelopoiesis or DC development was detected in either *boo/boo* or WT type co-cultures, with no production of lymphoid cells or DC subsets expressing markers like CD8^+^ or B220^+^ [42–44]. L-DC production was maintained by co-cultures established with both *boo/boo* and WT bone marrow for up to 6 months (data not shown), which was consistent with all previous studies on *in vitro* hematopoiesis with L-DC production [26, 34, 42].

## Discussion

In this paper, we used flow cytometry to detail changes in hematopoiesis due to the *c-Myb*^*E308G*^ mutation in mice. Changes in the representation of dendritic and myeloid cells present in blood and spleen were compared with changes in the production of progenitors of these lineages in bone marrow. Investigations were aimed at detecting coincident changes which supported a common role for the *c-Myb*^*E308G*^ mutation in cell lineage development. In chimera studies, the effect of the *c-Myb*^*E308G*^ mutation was also found to be intrinsic to hematopoietic cells and not somatic cells, which was consistent with the known function of *c-Myb* in controlling hematopoiesis in bone marrow. A similar finding was also reported for the *c-Myb*^*M303V*^ mutant strain [9]. The interpretation of subset data *in vivo* is not straightforward and has to consider compensatory effects when one subset is repressed and others increase to fill tissue spaces. In chimeras, there is also the effect of competition between *boo/boo* and WT progenitors to seed niches for hematopoiesis.

**Fig 8:**
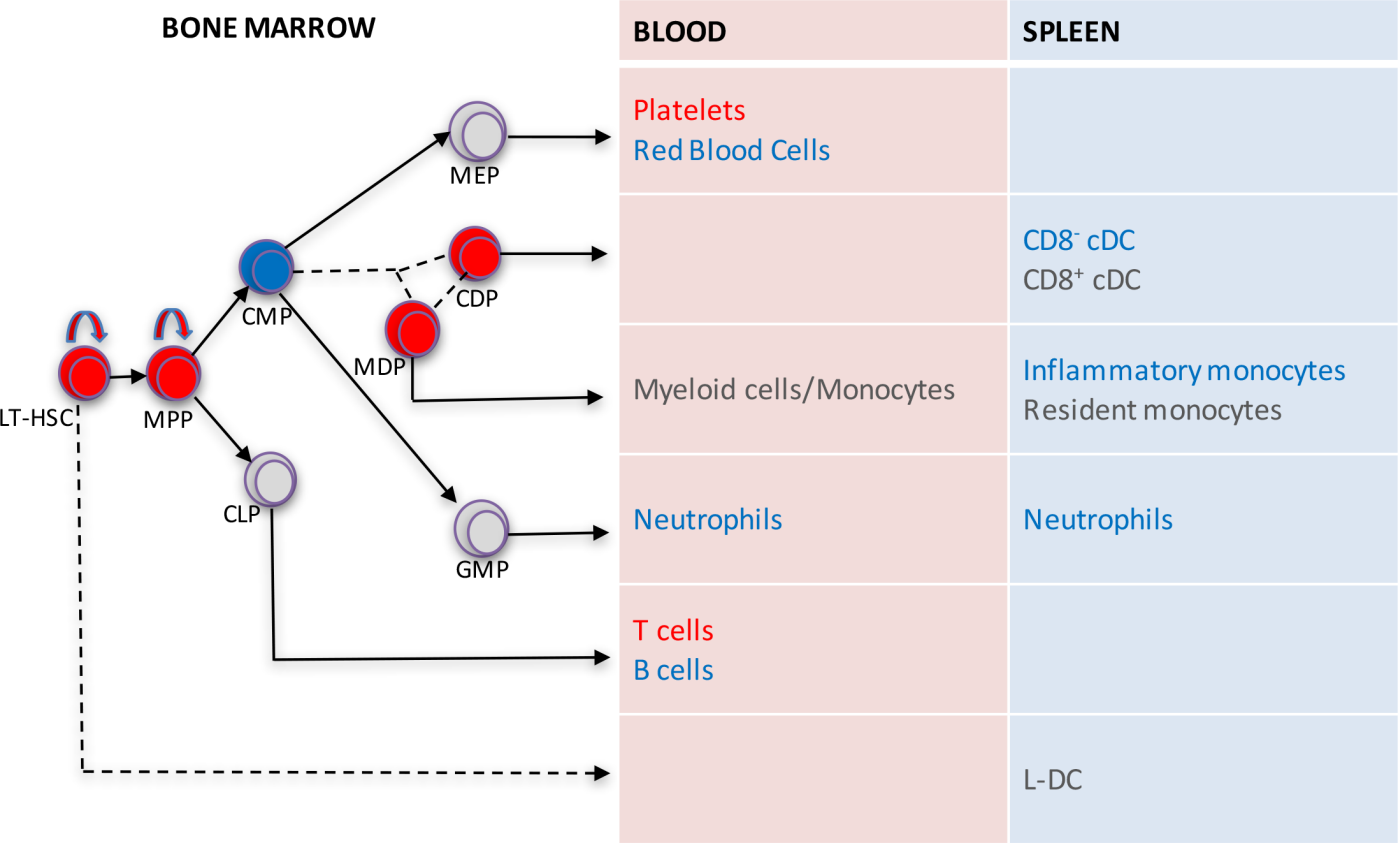
Changes in hematopoiesis attributable to the *c-Myb*^*308G*^ mutation. The summary diagram combines analysis of stem/progenitor subsets in mutant mouse bone marrow, with subset analysis of mature cell subsets in blood and spleen of chimeras reconstituted with mutant fetal liver cells and analysed at 16 weeks after reconstitution. Significant changes in cell production in relation to wild type mice or wild type control chimeras are shown in red indicating increase, or blue indicating a decrease, in cell production. Subsets unchanged in mutant and wild type are shown in grey. The diagram is not a complete lineage map since only subsets analysed are shown. These include: LT-HSC, longterm hematopoietic stem cell; MPP, multipotential progenitor; CMP, common myeloid progenitor; CLP, common lymphoid progenitor; MDP, myeloid dendritic progenitor; CDP common dendritic progenitor; MEP, myeloid/erythroid progenitor; GMP, granulocyte/macrophage progenitor. The lineage relationship between the MDP subset and CMP and CDP is shown as a dashed line since it is still in doubt [45]]. Similarly, the derivation of L-DC directly from bone marrow HSC and MPP is shown as a dashed line, since this has only been confirmed *in vitro* [28].

The *c-Myb*^*308G*^ mutation was found to impact hematopoietic cell development in a complex way, mapping to early HSPC expansion with less development of some mature cell types including CD8^-^ cDCs, inflammatory monocytes, and neutrophils. This mutation had little effect on the development of CD8^+^ cDCs, resident monocytes, and L-DCs. It clearly showed differential effects on inflammatory versus resident monocytes, and on L-DCs versus CD8^-^ cDCs. The *c-Myb*^*E308G*^ mutation therefore served to distinguish the lineage origin of these two monocyte subsets, as well as some DC subsets. A summary diagram of the effects detected here is shown in Fig 8.

Mutant *c-Myb*^*308G*^ mice were also useful for distinguishing the lineage origin of dendritic and myeloid subsets in spleen [10, 46]. Recent studies have indicated that some tissue macrophages in spleen develop from yolk sac-derived progenitors endogenous to spleen, while other monocytes/macrophages develop from bone marrow-derived progenitors [47]. Data shown here indicate at least two lineages of macrophages/monocytes since the production of inflammatory monocytes in spleen was reduced due to mutation in *c-Myb*, while production of resident monocytes was not. Resident monocytes may develop from progenitors which do not originate in bone marrow. The data are also consistent with L-DC development being *c-Myb*-independent, involving endogenous progenitors in spleen, perhaps of yolk sac origin, while CD8^-^ cDCs arise from *c-Myb*-dependent progenitors arising in bone marrow. This result is consistent with previous findings that L-DCs are developmentally distinct from cDCs, with only L-DC and not cDC arising in a Flt3-ligand- and GM-CSF-independent manner [48]. These findings support the possibility that L-DCs and resident monocytes in spleen arise from progenitors that are not bone marrow-derived and may be endogenous to spleen as yolk sac-derived hematopoietic progenitors. Whether L-DCs and resident monocytes are related in terms of lineage is currently unknown.

In terms of early hematopoiesis, our comprehensive analysis of *booreana* mice showed increased production of more primitive self-renewing HSPCs including subsets of LT-HSCs, ST-HSCs and MPPs in bone marrow, which was a result previously reported for fetal liver [12]. However, in contrast to fetal liver, the bone marrow of *booreana* mice showed no change in CMP and CLP populations. Since these are more committed hematopoietic progenitors, the question arises as to whether CLPs and CMPs in fetal liver have the same differentiative potential as their equivalents in adult bone marrow. Indeed, the *c-Myb*^*E308G*^ mutation appeared to enhance the self-renewal capacity of progenitors and inhibit their differentiative capacity since CMPs, GMPs and MEP were all reduced in number, with the greatest reduction being in GMPs. A reduction in myeloid lineage progenitors was also reflected by a decrease in the number of mature myeloid lineage cells including granulocytes and inflammatory monocytes (Figs 1 and 6).

In stromal co-cultures supporting *in vitro* hematopoiesis, L-DC have been shown to arise directly from early hematopoietic progenitors. This conclusion was reached in a study using *Ikaros*^*Plastic*^ homozygous mutant mice which live to only E15.5 but produce HSCs after E12.5 which lack self-renewal capacity, and no other hematopoietic progenitors [49]. While WT E14.5 progenitors seeded co-cultures and produced L-DC out to 28 days, fetal liver HSCs from E14.5 *Ikaros*^*Plastic*^ mutant mice were able to give short-term production of L-DC across days 7 and 14 of co-culture [50]. This result was interpreted to reflect direct differentiation of L-DCs from HSCs. L-DCs can also arise in stromal co-cultures from progenitors isolated from spleen [39–41], as well as HSC and MPP subsets sorted from fetal liver [26] and bone marrow [27, 28]. The increased numbers of LT-HSCs and MPPs in the bone marrow of *booreana* over WT mice needs to be considered in terms of L-DC development since both bone marrow-derived LT-HSCs and MPPs were previously shown to contain L-DC progenitors [28]. Increases of up to 8-fold for LT-HSCs and ~6-fold for MPPs in bone marrow suggest strong deregulation of hematopoietic development in *c-Myb*^*E308G*^ mutant mice. These changes, however, related to bone marrow specifically since L-DC numbers in spleens of mutants were found to be maintained at WT levels (Fig 6). This would be consistent with their *c-Myb* independent development from yolk sac-derived progenitors present in spleen in the normal steady-state mouse.

An investigation into the development of DC lineages also showed increases of 4-5-fold in numbers of CDPs and MDPs in *booreana* over WT mice. In line with evidence that *c-Myb*^*E308G*^ imposes increased self-renewal of HSPCs, these two progenitor subsets now seem more aligned with the development of MPPs and LT-HSCs, and quite distinct from the more committed CMP and GMP populations. Indeed, this information is consistent with a separate dendritic lineage of cells, distinct from the myeloid lineage. It is possible that the MDP progenitor is therefore mixed and leads to the separate development of a self-renewing CDP, which then seeds the development of conventional and plasmacytoid DC, and a CMP that seeds development of monocytes/macrophages, granulocytes, and erythroid lineages. CDP and MDP are unique to bone marrow, while the subsequent development of cDCs occurs in spleen from incoming pre-cDCs [15]. As with L-DC development from LT-HSCs and MPPs, the increase in CDP (and MDP) numbers in bone marrow did not coincide with an increase in total cDC numbers (CD8^+^ and CD8^-^ subsets) in spleen. Other environmental or regulatory factors must therefore determine the population size of mature cDCs that develop in spleen.

Both the *c-Myb*^*E308G*^ mutant mouse studied here and the *c-Myb*^*M303V*^ mutant described by Sandberg *et al* [9] contain a mutation in the transactivating region of *c-Myb* which interacts with the coactivator P300. However, the two mutant strains do not give completely consistent phenotypes. There are effects common to the two strains, and some distinct effects. For example, LT-HSC are increased in number by ~10 fold in both mutants and this has been attributed to an increase in the autonomous proliferative capacity of mutant LT-HSC [9]. In both studies, CLP numbers remain almost unchanged, erythyrocytes are reduced by ~1.2 fold, platelets are increased by 2-fold, and B cell numbers are reduced by 4–5 fold. However, several distinct effects are seen. The *E308G* mutation studied here is associated with 4–5 fold increases in number of MDP and CDP, reduced numbers of CMP and GMP, neutrophils and inflammatory monocytes, and a small increase in T cell production. In contrast the *M303V* mutation shows reduced numbers of MEP and CMP, no change in GMP, MDP and CDP numbers, no change in neutrophils, basophils or monocytes, while T cell numbers are reduced dramatically by 4–5 fold. The two mutants differ most noticeably in terms of development of T cells versus myeloid cells. The two strains have however not been compared in the same experiment, and estimates of cell subsets will always be dependent on the criteria or antibodies used to detect cells.

Both the *c-Myb*^*M303V*^ and the *c-Myb*^*E308G*^ mutant mouse strains contain a mutation in the transactivating region of c-MYB which interacts with the coactivator P300. In the case of homozygous *c-Myb*^*M303V*^ mice, Sandberg *et al* [9] have shown transactivation capacity to be reduced to ~50% of wild type animals. This leaves open the possibility that the *E308G* mutation may have different capacity to bind P300, and different transactivation capacity, which could differentially affect the production of cells in any hematopoietic pathway dependent on *c-Myb*. Indeed *c-Myb* is known to have multiple effects acting on both stem cells and later stages of hematopoiesis [12].

## Conclusion

Despite major disturbance among the early hematopoietic progenitors in bone marrow, and also among mature myeloid and erythroid cell types, *booreana* mice displayed no disturbance in the presence or prevalence of L-DCs in spleen. This result was consistent with their development from progenitors that were *c-Myb*-independent and were not bone marrow-derived hematopoietic progenitors.

## Supporting information

S1 Fig Schematic showing the production of chimeras using *booreana* and WT fetal liver. Lethally irradiated (9.5 Gray) CD45.1^+^ host mice were transplanted with either 1 x 10^6^ (1M) or 2 x 10^5^ (200K) fetal liver cells from either *boo/boo* or WT mice of CD45.2^+^ origin. In addition, 2 x 10^6^ (2M) supporting BM cells of F_1_ (CD45.1 x CD45.2) origin were given to each host. Flow cytometric profiles used to distinguish host and donor origin cells are shown.
(PPTX)

S2 Fig Flow cytometric gating strategy used to identify mature progeny of donor cells. Donor cell reconstitution was assayed by staining TER119^-^ CD45.2^+^ peripheral blood cells for markers of mature hematopoietic cells (CD3, B220, Mac1, Gr-1) monthly until to 16 weeks post transplantation.
(PPTX)

S3 Fig In vivo reconstitution potential of *booreana* fetal liver HSC. Mouse chimeras were generated as described in S1 Fig to investigate the *in vivo* reconstitution ability of *booreana* (*boo*) versus wild-type (WT) fetal liver. The lineage composition of the reconstituted compartment of peripheral blood from donor 1 x 10^6^ (1M) or 2 x 10^5^ (200K) fetal liver cells from either *boo/boo* or WT mice of CD45.2^+^ origin is shown as a kinetic (monthly) measure post-transplantation.
(PPTX)
