# Supplementary material for: Impact of the c-MybE308G mutation on mouse myelopoiesis and dendritic cell development
Source: PLoS One. 2017 Apr 26;12(4):e0176345. doi: 10.1371/journal.pone.0176345 (PMC5405991; doi:10.1371/journal.pone.0176345)
Supplement: S1 Fig — Lethally irradiated (9.5 Gray) CD45.1+ host mice were transplanted with either 1 x 10^6^ (1M) or 2 x 10^5^ (200K) fetal liver cells from either boo/boo or WT mice of CD45.2+ origin. In addition, 2 x 10^6^ (2M) supporting BM cells of F1 (CD45.1 x CD45.2) origin were given to each host. Flow cytometric profiles used to distinguish host and donor origin cells are shown. (PPTX) [file pone.0176345.s001.pptx]

## Slide 1
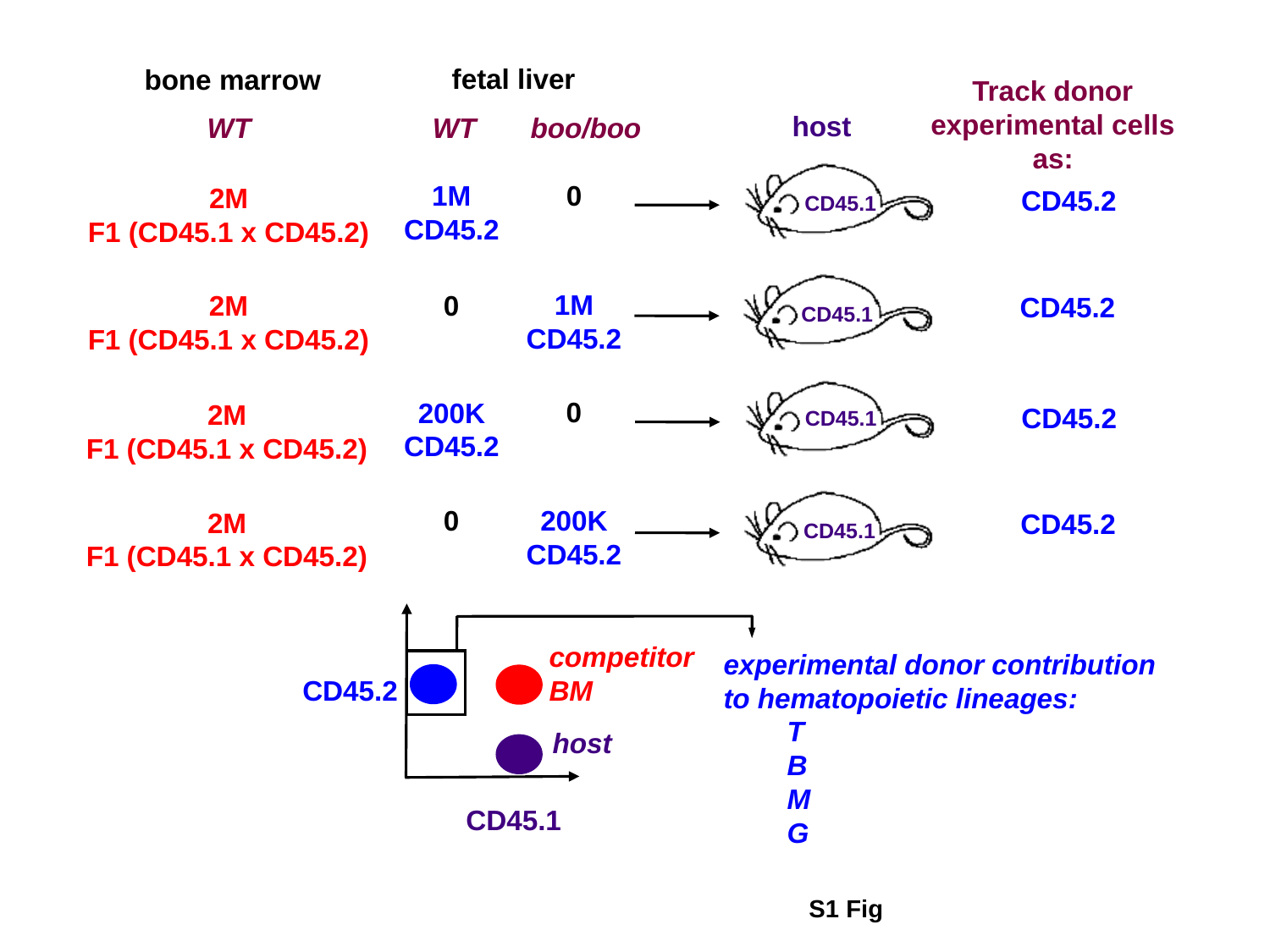

fetal liver
bone marrow
Track donor experimental cells as:
host
WT
boo/boo
WT
0
1M
CD45.2
2M
F1 (CD45.1 x CD45.2)
CD45.2
CD45.1
1M
CD45.2
0
2M
F1 (CD45.1 x CD45.2)
CD45.2
CD45.1
0
200K
CD45.2
2M
F1 (CD45.1 x CD45.2)
CD45.2
CD45.1
200K
CD45.2
0
2M
F1 (CD45.1 x CD45.2)
CD45.2
CD45.1
competitor BM
experimental donor contribution to hematopoietic lineages:
T
B
M
G
CD45.2
host
CD45.1
S1 Fig
