# Supplementary figures and images for: Impact of the c-MybE308G mutation on mouse myelopoiesis and dendritic cell development
Source: PLoS One. 2017 Apr 26;12(4):e0176345. doi: 10.1371/journal.pone.0176345 (PMC5405991; doi:10.1371/journal.pone.0176345)

## Slide 1
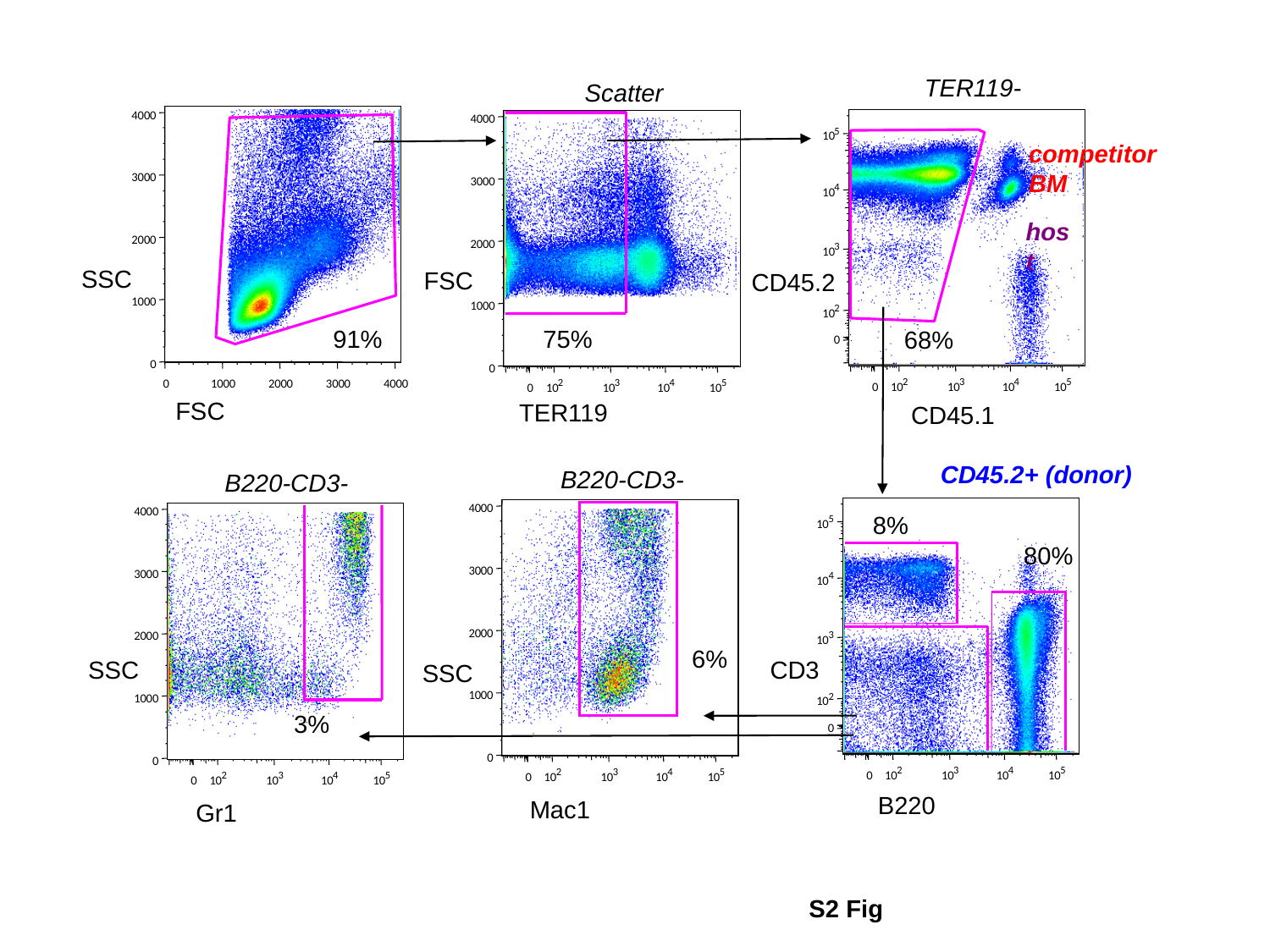

TER119-
Scatter
4000
3000
2000
FSC
1000
75%
0
2
3
4
5
0
10
10
10
10
TER119
4000
5
10
competitor BM
3000
4
10
host
2000
3
10
SSC
CD45.2
1000
2
10
CD45.2+ (donor)
8%
5
10
80%
4
10
3
10
2
10
0
2
3
4
5
0
10
10
10
10
B220
CD3
91%
68%
0
0
2
3
4
5
0
1000
2000
3000
4000
0
10
10
10
10
FSC
CD45.1
B220-CD3-
4000
3000
2000
6%
1000
0
2
3
4
5
0
10
10
10
10
Mac1
SSC
B220-CD3-
4000
3000
2000
SSC
1000
3%
0
2
3
4
5
0
10
10
10
10
Gr1
S2 Fig

Supplement: S2 Fig — Donor cell reconstitution was assayed by staining TER119- CD45.2+ peripheral blood cells for markers of mature hematopoietic cells (CD3, B220, Mac1, Gr-1) monthly until to 16 weeks post transplantation. (PPTX) [file pone.0176345.s002.pptx]

## Slide 1
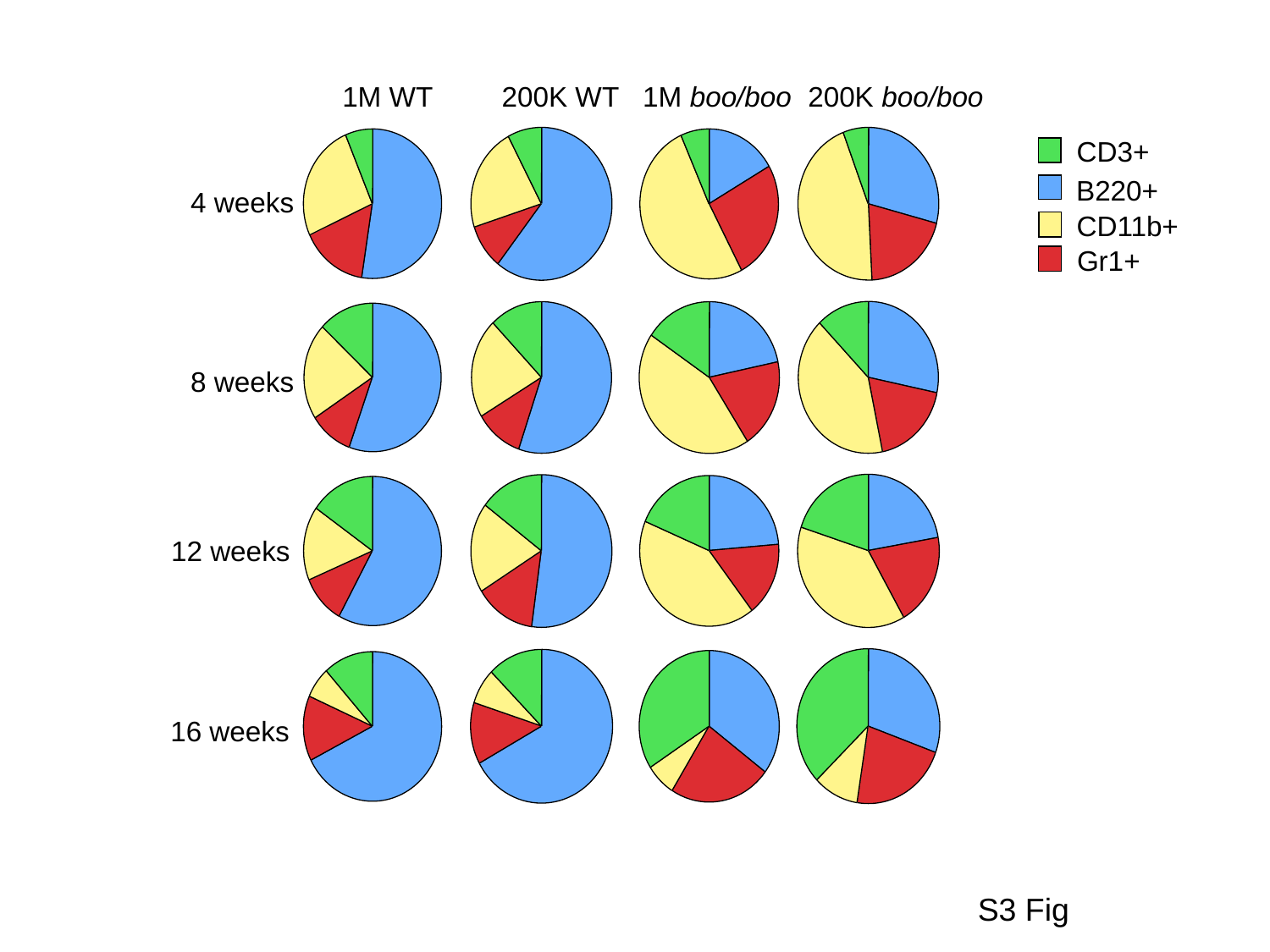

1M WT
4 weeks
200K boo/boo
200K WT
1M boo/boo
CD3+
B220+
CD11b+
Gr1+
8 weeks
12 weeks
16 weeks
S3 Fig

Supplement: S3 Fig — Mouse chimeras were generated as described in S1 Fig to investigate the in vivo reconstitution ability of booreana (boo) versus wild-type (WT) fetal liver. The lineage composition of the reconstituted compartment of peripheral blood from donor 1 x 10^6^ (1M) or 2 x 10^5^ (200K) fetal liver cells from either boo/boo or WT mice of CD45.2+ origin is shown as a kinetic (monthly) measure post-transplantation. (PPTX) [file pone.0176345.s003.pptx]
